# The molecular mechanism responsible for HbSC retinopathy may depend on the action of the angiogenesis-related genes *ROBO1* and *SLC38A5*

**DOI:** 10.3389/ebm.2024.10070

**Published:** 2024-07-24

**Authors:** Sueli Matilde da Silva Costa, Mirta Tomie Ito, Pedro Rodrigues Sousa da Cruz, Bruno Batista De Souza, Vinicius Mandolesi Rios, Victor de Haidar e Bertozzo, Ana Carolina Lima Camargo, Marina Gonçalves Monteiro Viturino, Carolina Lanaro, Dulcinéia Martins de Albuquerque, Amanda Morato do Canto, Sara Teresinha Olalla Saad, Stephanie Ospina-Prieto, Margareth Castro Ozelo, Fernando Ferreira Costa, Mônica Barbosa de Melo

**Affiliations:** ^1^ Center for Molecular Biology and Genetic Engineering, State University of Campinas—UNICAMP, Campinas, Brazil; ^2^ Centro de Hematologia e Hemoterapia, Universidade Estadual de Campinas—UNICAMP, Campinas, Brazil; ^3^ Departamento de Medicina Translacional, Faculdade de Ciências Médicas, Universidade Estadual de Campinas—UNICAMP, Campinas, Brazil

**Keywords:** proliferative sickle cell retinopathy, endothelial colony forming cells, transcriptome, differentially expressed genes, angiogenesis

## Abstract

HbSC disease, a less severe form of sickle cell disease, affects the retina more frequently and patients have higher rates of proliferative retinopathy that can progress to vision loss. This study aimed to identify differences in the expression of endothelial cell-derived molecules associated with the pathophysiology of proliferative sickle cell retinopathy (PSCR). RNAseq was used to compare the gene expression profile of circulating endothelial colony-forming cells from patients with SC hemoglobinopathy and proliferative retinopathy (n = 5), versus SC patients without retinopathy (n = 3). Real-time polymerase chain reaction (qRT-PCR) was used to validate the RNAseq results. A total of 134 differentially expressed genes (DEGs) were found. DEGs were mainly associated with vasodilatation, type I interferon signaling, innate immunity and angiogenesis. Among the DEGs identified, we highlight the most up-regulated genes *ROBO1* (log2FoldChange = 4.32, FDR = 1.35E-11) and *SLC38A5* (log2FoldChange = 3.36 FDR = 1.59E-07). *ROBO1*, an axon-guided receptor, promotes endothelial cell migration and contributes to the development of retinal angiogenesis and pathological ocular neovascularization. Endothelial *SLC38A5,* an amino acid (AA) transporter, regulates developmental and pathological retinal angiogenesis by controlling the uptake of AA nutrient, which may serve as metabolic fuel for the proliferation of endothelial cells (ECs) and consequent promotion of angiogenesis. Our data provide an important step towards elucidating the molecular pathophysiology of PSCR that may explain the differences in ocular manifestations between individuals with hemoglobinopathies and afford insights for new alternative strategies to inhibit pathological angiogenesis.

## Impact statement

Here we described genes differentially expressed identified in circulant endothelial progenitor cells from SC patients with proliferative retinopathy and their respective functional pathways. Our data, potentially related with sickle cell retinopathy, provide favorable evidence for further studies about molecular mechanisms involved in this complication. Among DEGS identified we highlight the up-regulated genes *ROBO1* and *SLC38A5.* Both genes are known to play important role in angiogenesis. We believe that these genes deserve more attention as an alternative strategy to inhibit pathological angiogenesis, hallmark of PSCR. The identification of new targets to anti-angiogenic therapy is of great interest, particularly in individuals who are resistant to current therapies.

## Introduction

Sickle cell retinopathy (SCR) is the major ocular complication of sickle cell disease (SCD), occurring in non-proliferative and proliferative forms. Proliferative sickle cell retinopathy (PSCR) can lead to some degree of visual loss in 5%–20% of affected eyes [1]. This complication results from the aggregation of sickle-shaped red blood cells in the retinal microcirculation, leading to peripheral arterial occlusion, thrombosis, and ischemia, which may be precipitated by hypoxia, acidosis, and hyperosmolarity. Besides being a red blood cell disorder, the pathophysiology of vasoocclusion and tissue ischemia involves inflammation, endothelial cell activation, the presence of procoagulant and proangiogenic molecules and vasculopathy [2].

Vascular occlusion causes ischemic events leading to the release of angiogenic mediators, which may result in retinal neovascularization. The hallmark of PSCR is sea fan formations, new vessels that often assume a frond-like configuration and occur when the neovascularization grows anteriorly from the vascular to avascular retina. These structures may spontaneously regress (20%–60%) by autoinfarction [3]. However, neovascularization can adhere to the inner surface of the retina and the outer surface of the vitreous [4]. Since new blood vessels lack tight junction proteins, plasma can leak into surrounding tissue, including the vitreous, leading to vitreous degeneration and hemorrhage. In addition, the degraded vitreous can contract and pull on the retina, resulting in retinal detachment. Traction retinal detachment involving the macula results in severe vision loss [4].

Although sickle cell anemia (HbSS genotype) has the most severe systemic manifestations, patients with hemoglobin SC disease (HbSC genotype) have the most severe ocular manifestations and an earlier age of onset [2]. The reasons for this discrepancy are unknown. Blood hyperviscosity [5], lower Hb F levels [6] and lower plasma thrombospondin (TSP) levels [7], usually reported in HbSC patients, have been hypothesized to increase the risk for the development of PSCR in these patients.

Retinal neovascularization plays a crucial role in PSCR as well as in other ischemic retinopathies, such as retinopathy of prematurity (ROP) and proliferative diabetic retinopathy (PDR). Many studies in mouse models have shown that there is increased activity of hypoxia-inducible factor 1 (HIF-1) in ischemic retinopathies, causing up-regulation of several hypoxia-regulated genes associated with angiogenesis, including vascular endothelial growth factor (VEGF), essential regulator of angiogenesis and vascular permeability; angiopoietin 2 (ANGPT2), placental growth factor (PlGF), and stromal derived growth factor-1 (SDF-1) among others [4, 8, 9]. Furthermore, extra-cellular signaling pathways such as the Ephrin/Eph receptor, Netrin/UNC receptor and Robo/Slit are involved in the formation of new vessels [10].

Angiogenesis, the growth of new vessels from pre-existing vessels by sprouting endothelial cells, requires the coordinated action of a variety of angiogenic stimulators and inhibitory factors. Although a mouse model of ischemic retinopathy has widely contributed to elucidate the basis of the molecular pathogenesis involved in retinal neovascularization, the literature is still scarce regarding the molecular aspects of PSCR. The aim of the present study was to identify differences in the expressions of molecules produced by endothelial cells that may be associated with the clinical ocular heterogeneity among individuals affected by SC hemoglobinopathy. Identifying molecules associated with PSCR development may help to better elucidate pathogenic mechanisms underlying SCD-induced vision loss and establish new therapeutic targets.

We used RNA-Seq to compare the gene expression profiles of circulating endothelial colony-forming cells (ECFCs) from patients with SC hemoglobinopathy and proliferative retinopathy versus patients without retinopathy. Endothelial colony-forming cells (ECFCs), also called outgrowth endothelial cells or “late” endothelial progenitor cells (EPCs), are the most potent vascular reparative cells type among EPC. These cells are potentially capable of incorporating into foci of physiological or pathological neovascularization [11, 12]. Mouse model studies have provided evidence that ECFCs may play an important role in the regeneration of retinal vasculature and reduction of pathological angiogenesis in a model of ischemic retinopathy [13–15]. Furthermore, to elucidate molecular differences between genotypes, we investigated whether differential expression of the main genes detected in HbSC patients was also observed when HbSS patients, with and without PSCR, were compared. The results presented here, to the best of our knowledge, represent the most comprehensive analysis of comparative gene expression in human EPCs currently available.

## Materials and methods

### Patients

A total of 10 HbSC and 8 HbSS patients were recruited from the Hematology and Hemotherapy Center, University of Campinas-UNICAMP (Campinas, São Paulo, Brazil). Among the HbSC patients, six had PSCR (Group 1) and four had no retinopathy (Group 2). Among the HbSS patients, four had PSCR (Group 3) and no pathological ophthalmic signs were observed in four (Group 4). Hemoglobinopathy diagnosis was performed through clinical and laboratorial data. Demographic characteristics and hematological parameters were collected (Supplementary Tables S1, S2), as well as clinical data and information on drug therapy (Supplementary Tables S3, S4). It should be emphasized that, in SCD patients under regular blood transfusion therapy (such as in patients with stroke), the peripheral blood samples for isolation of ECFCs were collected immediately before transfusion, at least 15 days after the previous transfusion. All SCD patients in this study were not on hydroxycarbamide (also known as hydroxyurea) therapy or any other disease modifying therapy. After ophthalmic examinations, peripheral blood samples were collected and ECFCs cultures were carried out immediately. Exclusion criteria were malignancy, diabetes mellitus, pregnancy, painful crisis within the 2 weeks before the time of ocular examination, ocular media opacities that precluded fundoscopic examination, hydroxycarbamide therapy and previous eye surgery.

All patients provided written informed consent and the study was approved, in accordance with national guidelines, by the University Ethics Committee.

### Ophthalmologic evaluation

All patients in this study underwent a complete ophthalmic examination, which included anterior biomicroscopy, fundus biomicroscopy using 78-diopter lens, indirect fundoscopy with a 20-diopter lens, color fundus photography and fluorescein angiography. The patients were examined by the same ophthalmologist at the Ophthalmology Department of the University of Campinas. In the PSCR groups (1 and 4) patients in stages III, IV and V, according to Goldberg’s classification [16], were included with the following ophthalmic features: neovascularized sea fan that occurs at the boundary between the non-vascularized and vascularized zones of the retinal periphery (Stage III), presence of vitreous hemorrhage (Stage IV), tractional or rhegmatogenous retinal detachment (Stage V).

### ECFC culture

Cultures of ECFCs were established from peripheral blood samples, according to previously described methods [17, 18]. Peripheral blood (45 mL) was collected in a heparinized solution and samples were processed immediately. The anticoagulated blood was diluted in 2 parts of phosphate-buffered saline solution (PBS) and then added to an equivalent volume of Ficoll-Paque PLUS (GE Healthcare, Uppsala, SE) before centrifugation at 317g for 30 min at room temperature. Mononuclear cells were isolated and washed 3 times with EBM-2 medium (Lonza, Walkersville, MD, United States) and resuspended in EBM-2 medium (Lonza, Walkersville, MD, United States) containing EGM-2 (Endothelial Cell Growth Medium-2) BulletKit™, 10% additional fetal bovine serum (Invitrogen, Carlsbad, CA, United States), 1% penicillin/streptomycin (Invitrogen, Carlsbad, CA, United States) and 1% L-glutamine (Gibco, Life Technologies, Carlsbad, California, United States). Approximately 7 × 10^6^ cells were seeded onto twelve-well flat-bottom tissue culture plates precoated with type 1 rat-tail collagen (Sigma-Aldrich, Saint Louis, MO, United States) and cultured in a humidified incubator at 5% CO_2_. After an incubation time of 7–21 days, ECFCs were identified by their typical cobblestone morphology and were evaluated for surface marker expression with a FACSCalibur (BD Bioscience, San. Jose, CA, United States). Results were analyzed using the FlowJo software (Tree Star Inc.). ECFCs were characterized by positive staining for endothelial markers CD31, CD144, CD146, VEGF/KDR; negative or low for endothelial activation antigen, CD34; negative for the myeloid cell marker, CD45, and for the endothelial progenitor marker, CD133.

### RNA extraction

After reaching 80%–90% confluence, the ECFCs were harvested using 0.025% trypsin-EDTA. Total RNA was extracted from cells using Trizol Reagent (Ambion Life Technologies, Carlsbad CA, United States), and a commercial RNeasy mini Kit (Qiagen GmbH, Hilden, Germany), according to the manufacturer’s instructions. RNA was treated with DNAse I (Life Technologies, Carlsbad, CA, United States) to remove remaining genomic DNA. The quantity and purity of the extracted RNA were measured using a NanoDrop 2000 spectrophotometer (Thermo Scientific), and RNA integrity was determined using the Bioanalyzer 2100 system (Agilent Technologies).

### RNA sequencing and differential gene expression analysis

RNA sequencing (RNA-Seq) was performed on the ECFC samples from SC patients, comprising five patients from Group 1 and three patients from Group 2. RNA libraries were prepared using Illumina TruSeq RNA-Seq v2 kit (Illumina, San Diego, CA, United States) according to manufacturer’s protocol. After quality control by Agilent Bioanalyzer (Agilent, Santa Clara, CA), the libraries were submitted to paired-end 100 bp high output sequencing on a HiSeq 2500 instrument. Read quality was assessed by FastQC v0.11.5 [19] and aligned to human genome assembly (GRCh38.88) using STAR version 2.5.2 [20]. Only those reads uniquely mapped to the reference genome were subsequently analyzed.

Differential gene expression analysis was performed using the DESeq2 [21] R package. The FeatureCounts algorithm from Rsubread was used to generate count matrices from reads aligned to the genome [22]. To visualize the overall gene expression levels, a Volcano plot was created with log2FoldChange score and log10 *p*-values through to the EnhancedVolcano package (v1.2.0) [23]. The changes in gene expression levels were considered significant when log2FoldChange were ≥| +1 ≤|−1|, and statistical test values (false discovery rate—FDR—adjusted *p*-value) were lower than 0.1. Significance threshold <0.1 was set for Volcano plot to capture highly abundant marginal changes in gene expression. ClustVis [24] was used to build the heatmaps using Pearson’s correlation coefficient. *PCAtools* (Principal Component Analysis) [25] of RStudio were used. The relationships between the molecular pathways and ten DEGs selected for validation demonstrated in an alluvium diagram, were performed on the Sankeymatic platform.^1^

### Gene ontology (GO) and protein-protein interaction (PPI) network analysis

The PANTHER v14.1 online tool was used to perform enrichment analysis for GO Biological Processes (Fisher’s exact test; FDR ≤0.05). All expressed genes (count >5) were used to build the reference list (background) and DEGs with FDR <0.1 were included in the list of analyzed genes.

Cytoscape Network Analysis (v3.8.1) was used to construct and visualize the protein-protein interaction [26]. We used StringApp [27] to search for interactions using UNIPROT IDs [28] of DEGs. The parameters used were confidence score cutoff: 0.4, maximum additional interactors = 0 and load enrichment data. Non-coding genes were excluded before loading the resulting 123 DEGs to StringApp. Additionally, to identify the protein clusters from the overall protein interaction network generated by STRING, we used Markov clustering (MCL) [29] (Granularity parameter, inflation value = 4). Functional enrichment for each cluster and enrichment map (only the gene ontology results) were performed using the following parameters: connectivity cutoff (Jaccard similarity) = 0.4, node cutoff: q-value = 0.05, Style: Q-value FDR and Radial Heat Map. Additionally, we used the Cytohubba plugin to compute different scores for protein nodes and verify the most hub gene [30]. Parameters of cytoHubba were set as follows: Hubba nodes = top 1 node ranked by Degree, display options = check the first-stage nodes and display the shortest path.

### Validation by RT-PCR and expression level analysis of DEGs in HbSS patients

The quantitative real‐time polymerase chain reaction (qRT‐PCR) was used to validate the relative expression patterns obtained by RNA‐Seq. Ten genes with log2 fold change ≥| +1 ≤|−1| and FDR ≤ 0.05 were selected: five up-regulated genes, *ROBO1*, *SLC38A5, NNAT, PROX1, SEMA3B* and five down-regulated genes, *CCL2*, *CXCL10*, *GBP5*, *TGFB2* and *OASL*. These genes were selected from functional enrichment analysis due to their association with angiogenesis (FDR 1.59E-02), cell migration (FDR 1.10E-02), cell adhesion (FDR 5.77E-04), immune response (FDR 2.44E-03) and type I interferon signaling pathway (FDR 6.24E-03). To evaluate whether differential expression identified in HbSC patients was also present in HbSS patients, relative expression of these genes was also evaluated in an RNA sample of ECFCs from HbSS patients with (n = 4) and without (n = 4) retinopathy.

Complementary DNA (cDNA) was synthesized from 1 μg of RNA utilizing the RevertAid First Strand cDNA Synthesis Kit (Fermentas), according to the manufacturer’s instructions. The cDNA was used as template for real-time qRT-PCR analysis using SYBR green (Applied Biosystems, United States) based detection in an ABI StepOne Plus equipment (Applied Biosystems, Carlsbad, CA). Primer sequences, annealing temperatures and product sizes are provided in Supplementary Table S5. Melting curves were examined to ensure single products. Briefly, the PCR amplification was carried out in a 12 μL reaction volume containing 150 or 300 nM specific primers (3 μL), 10 ng cDNA (3 μL), and 6 μL SYBR Green PCR Master Mix (Applied Biosystems, United States). All samples were run in triplicate with the following cycling conditions: 1 cycle of 95°C for 10 min, 40 cycles of 95°C for 15s, and 40 cycles of 60°C for 1 min. Results were quantified using the “delta-delta Ct” method and normalized to the constitutive genes *ACTB* and *GAPDH* transcript levels. Relative expression for each sample was calculated using the standard equation [2^(−ΔCt)^], as previously described by Schmittgen [31]. Delta CT values between genes of interest and GAPDH were analyzed for statistical significance using a t-test by GraphPad Prism version 5.00 for Windows (GraphPad Software, La Jolla, CA, United States).

## Results

In the present study, RNA-Seq was used to characterize the ECFC transcriptomes of SC patients with (5) and without PSCR (3). After sequencing, RNAseq produced an average of 70 million 101-base pair paired-end reads per library. GC content was approximately 49%. A minimum of 88% of the bases reached a Q score of 30 (Q30), indicating low probability that a base was erroneously sequenced. A high number of reads (85%–94%) mapped exclusively to the human genome. Raw Illumina RNA-Seq FASTQ files are available in the Gene Expression Omnibus (GEO) database (Accession Number: GSE240446). All transcripts found in the comparison of the group 1 versus group 2 (26,299 variables) were depicted in a volcano plot (See Supplementary Figure S1).

### Identification of DEGs

**FIGURE 1:**
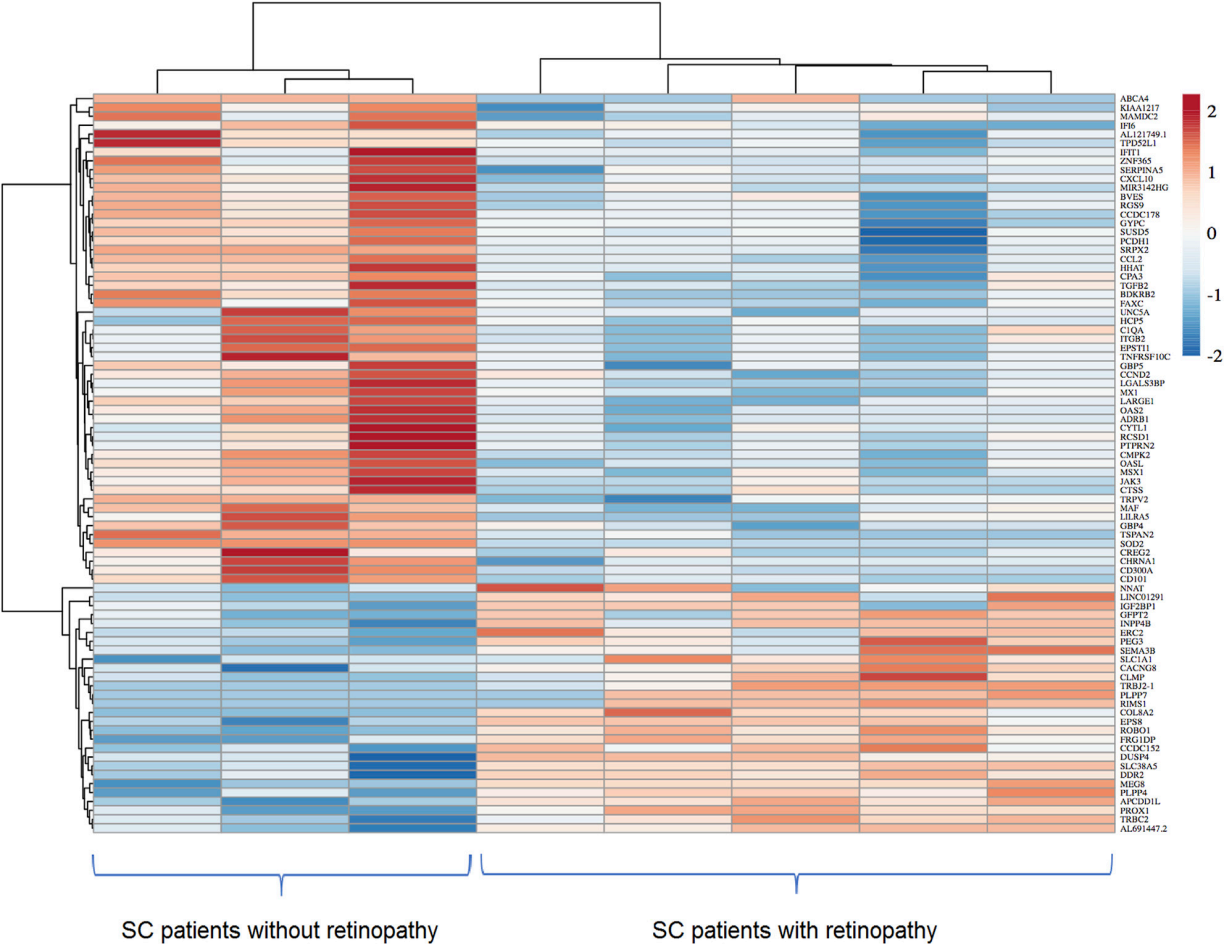
Heatmap showing differentially expressed genes identified after the comparison of ECFC transcriptomes of SC patients with and without PSCR. Differential expression threshold criteria of log2FoldChange ≥| +1 ≤|−1| and padj value < 0.05 were applied. Columns represent different samples, whereas rows represent differentially expressed genes. The intensity of the color was used to indicate the level of gene expression. The highest expression is represented by a darker red and the lowest expressions by a darker blue.

**FIGURE 2:**
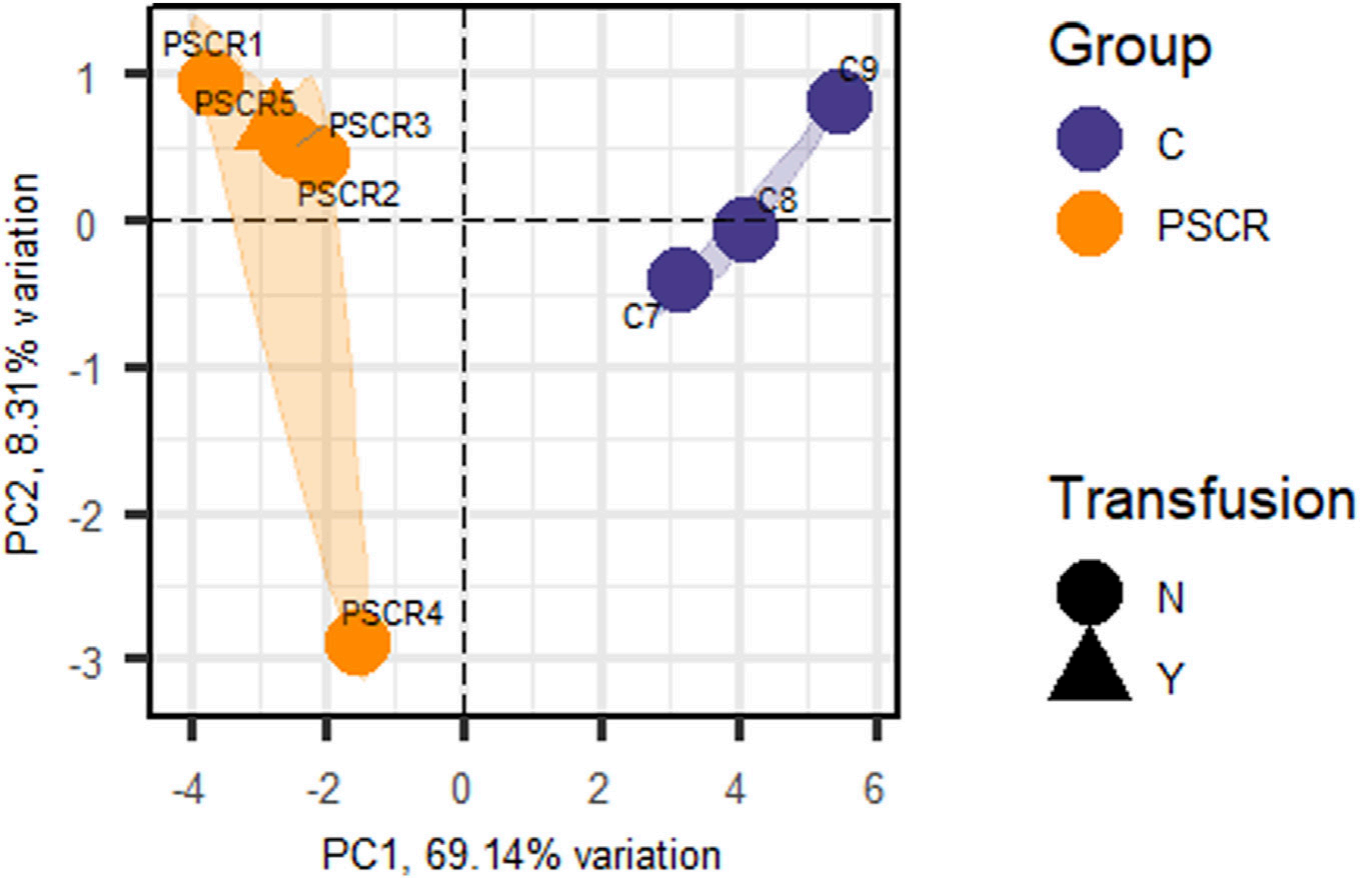
Principal Component Analysis (PCA) of the patients obtained from the DEGs (FDR <0.05) identified by RNA sequencing. Principal Component 1 (PC1) represents 69.14% of group variation, and Principal Component 2 (PC2), 8.31%. The triangular shape corresponds to individuals under transfusion treatment. PSCR, Proliferative sickle cell retinopathy; C, Controls; N, No; Y, Yes.

Considering statistically significant FDR < 0.1, a total of 134 DEGs were found when comparing SC patients with and without PSCR. Among these genes, 36 were up-regulated and 98 were down-regulated (Supplementary Tables S6, S7). Volcano plots and GO Functional Enrichment Analysis using Panther tools were generated from FDR < 0.1. A less conservative FDR threshold was used to illustrate the general gene expression pattern detected by DESeq2 and for a broader investigation of potential interactions. When a more stringent significance threshold (FDR<0.05) was used a total of 83 DEGs were found; 28 were up-regulated and 55 were down-regulated. A graphic overview of the differential status of gene expression is represented by the heatmap in Figure 1. A distinct clustering between groups with and without PSCR is also shown in the Principal Component Analysis (PCA) (Figure 2), in which the presence or absence of PSCR explains 69.14% of total group variance.

### GO functional enrichment analysis

**FIGURE 3:**
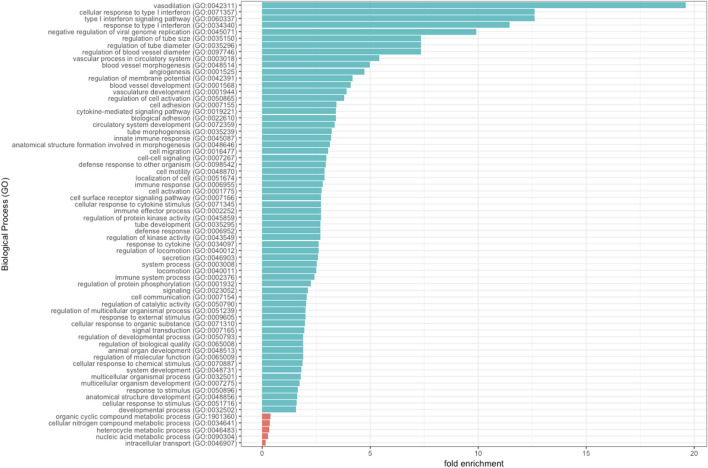
Significantly enriched GO terms of DEGs are shown. The 134 DEGs (36 up-regulated and 98 down-regulated) were enriched in biological process.

To gain further insights into the biological functions associated with genes that were differentially expressed in SC patients with PSCR, when compared with SC patients without retinopathy, we used the Panther online tool. GO terms were assigned to 98 down-regulated and 36 up-regulated DEGs. Biological process analysis includes significantly enriched gene ontology terms associated with vasodilation, such as regulation of tube size (FDR = 3.51e-02), regulation of tube diameter (FDR = 3.56e-02), regulation of blood vessel diameter (FDR = 3.62e-02) and vascular process in circulatory system (FDR = 1.21e-02). DEGs are also enriched in terms associated with immune process such as cellular response to type I interferon (FDR = 4.87e-03), type I interferon signaling (FDR = 5.09e-03), response to type I interferon (FDR = 6.96e-03), negative regulation of viral genome replication (FDR = 3.55e-02). GO terms associated with angiogenesis as blood vessel morphogenesis (FDR = 1.64e-03) and vasculature development (FDR = 9.77e-03) are also represented (Figure 3).

### Protein-protein interaction (PPI) network analysis

**FIGURE 4:**
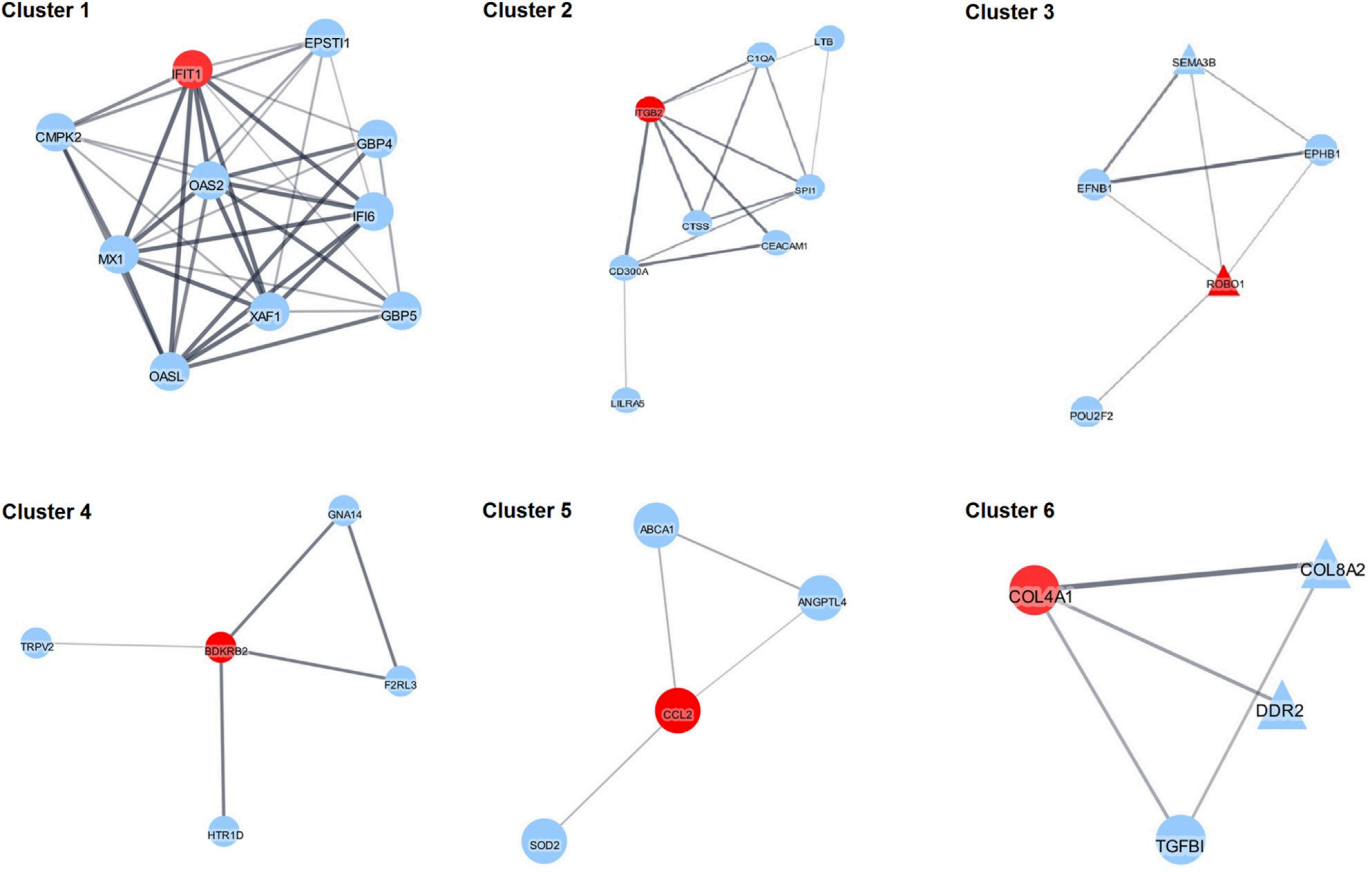
The five most significant clusters. Red nodes represent hub genes as inferred by Cytohubba. Triangles represent up-regulated and circles represent down-regulated DEGs.

To better understand interactions among the DEGs, PPI network analysis was performed. All the DEGs with corresponding known protein names were analyzed using the StringApp of the Cytoscape software. After filtering the nodes without connection, the main network was generated containing 64 nodes and 151 edges (Supplementary Figure S2). To group the proteins in the clusters based on their interactions from STRING we used the Cytoscape MCL plugin. Ten clusters with three nodes or more and six clusters with only two nodes were generated and the top six clusters are shown in Figure 4. Additionally, functional enrichment analysis and enrichment maps were performed for these six clusters (Supplementary Figure S3). In these clusters, six hub genes included *IFIT1*, *ITGB2*, *ROBO1*, *BDKRB2*, *CCL2* and *COL4A1* after analysis by the Cytohubba plugin. Pathway enrichment analysis showed that Cluster 1 is mainly associated with type I interferon signaling (FDR = 1.3e-10), innate immune response (FDR = 7.72E-9), response to other organism (FDR = 7.72E-9) and cellular response to cytokine stimulus (FDR = 7.46E-7). Cluster 2 is mainly relevant with tertiary granule (FDR = 3.35E-5), immune system process (FDR = 3.79E-5) and immune response (FDR = 6.24E-5). Cluster 3 is mainly associated with axon guidance (FDR = 4.31E-5), axon guidance receptor activity (FDR = 1.1E-4), cell development (FDR = 1.2E-4) and negative chemotaxis (FDR = 9.5E-4). Cluster 4 shows the main association with G protein-coupled receptor signaling pathway (FDR = 0.0075), regulation of anatomical structure size (FDR = 0.0075) and vasoconstriction (FDR = 0.0075). Cluster 5 shows that the most significant pathways are associated with homeostatic processes (FDR = 0.0202), regulation of endothelial cell apoptotic process (FDR = 0.0202), regulation of developmental processes (FDR = 0.0292). Cluster 6: the most significant terms are extracellular matrix organization (FDR = 1.17E-5), basement membrane (FDR = 1.93E-5), collagen-activated tyrosine kinase receptor signaling pathway (FDR = 1.2E-4) and angiogenesis (6.1E-4).

### Validation of expression level analysis of DEGs by qRT-PCR in HbSC and HbSS patients

**FIGURE 5:**
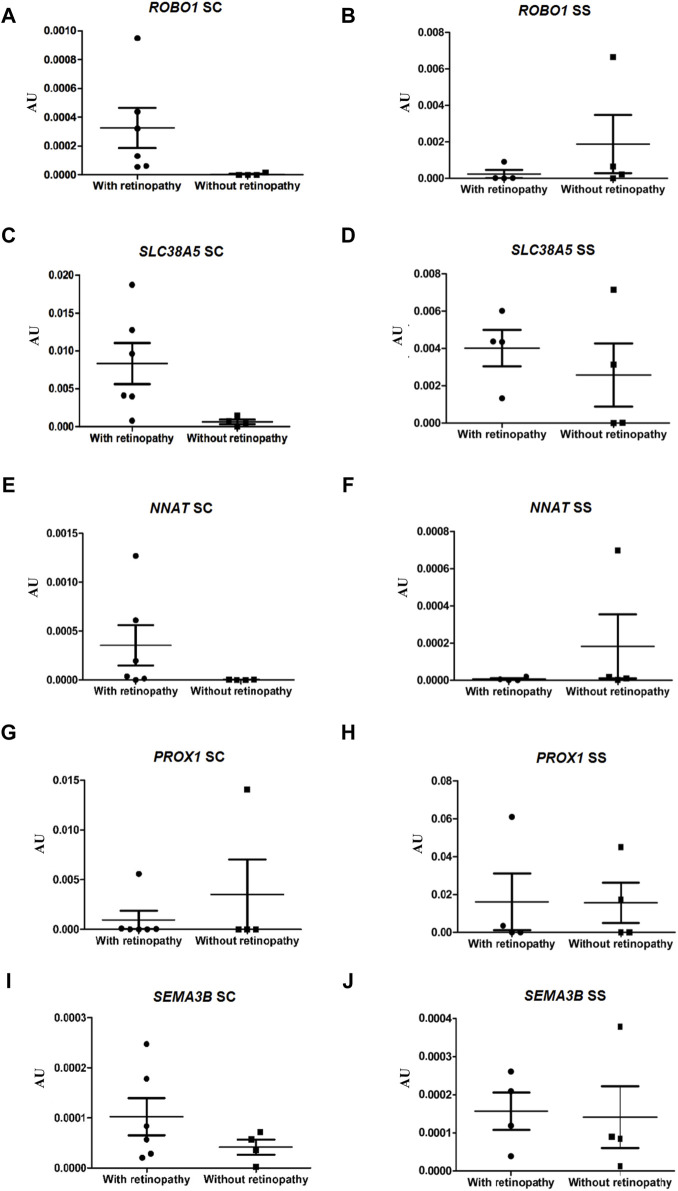
Validation of up-regulated genes, *ROBO1*, *SLC38A5*, *NNAT*, *PROX1* and *SEMA3B*, by RT-qPCR in HbSC patients and comparison of relative mRNA levels of the same genes in HbSS patients with and without retinopathy, mRNA expression was normalized to *ACTB* and *GAPDH* mRNA expression levels. Each dot represents the gene expression of an individual. AU (Arbitrary Unit). **(A,C,E,G,I)** HbSC patients. **(B,D,F,H,J)** HbSS patients.

**FIGURE 6:**
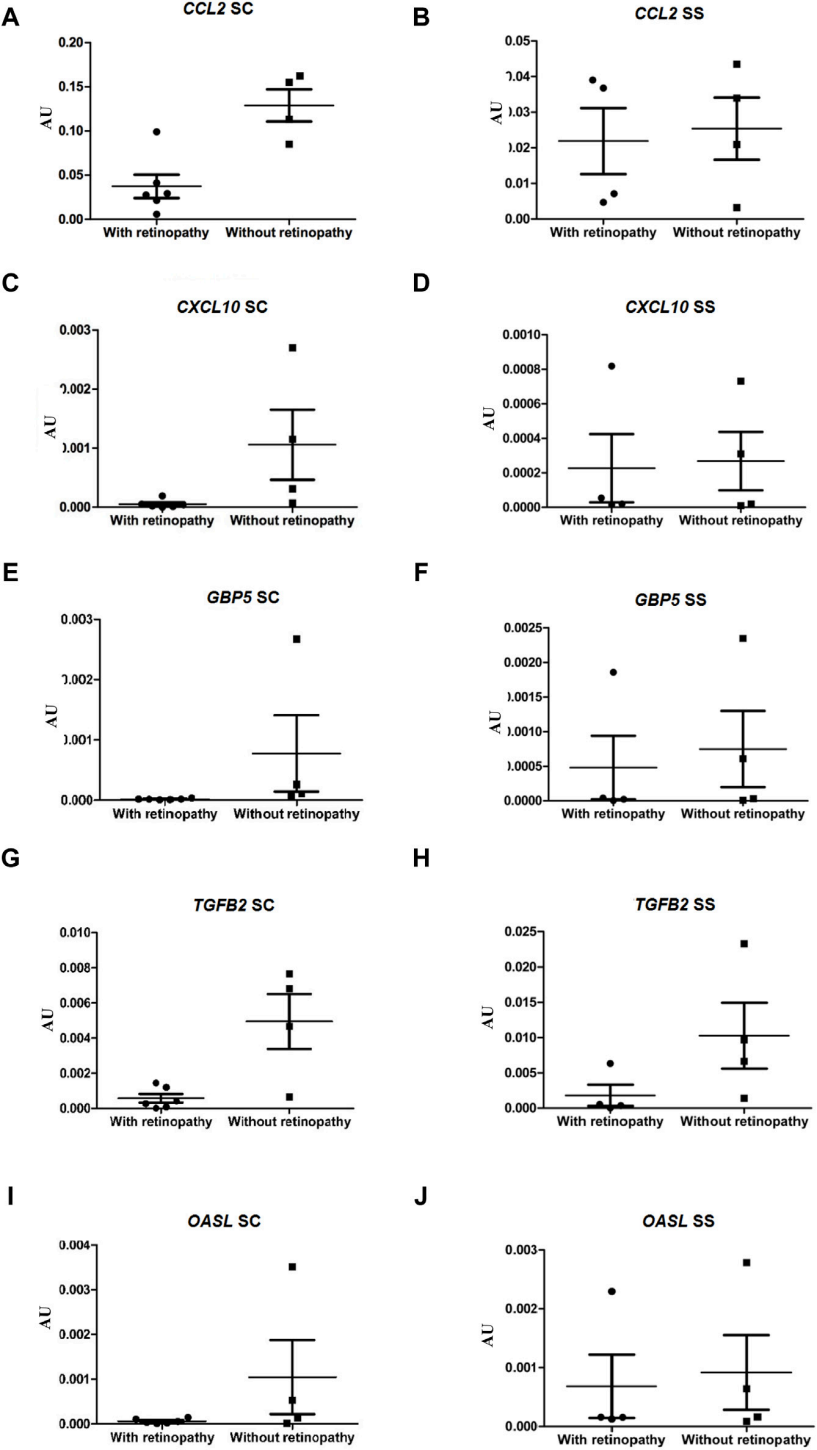
Validation of down-regulated genes, *CCL2*, *CXCL10*, *GBP5*, *TGFB2* and *OASL*, by RT-qPCR in HbSC patients and comparison of relative mRNA levels of the same genes in HbSS patients with and without retinopathy. mRNA expression was normalized to ACTB and GAPDH mRNA expression levels. Each dot represents a patient. AU (Arbitrary Unit). **(A,C,E,G,I)** HbSC patients. **(B,D,F,H,J)** HbSS patients.

To validate the RNA-seq data, the expressions of ten genes in the ECFCs from HbSC patients with and without retinopathy were verified by qRT-PCR. In the present study, we also investigated the expression levels of these same DEGs in groups III and IV (HbSS patients with and without retinopathy, respectively). The expressions of the *ROBO1*, *SLC38A5* and *NNAT* genes were significantly higher in HbSC patients with PSCR, *p*-value <0.05. However, *NNAT* failed to achieve this threshold value after Benjamini Hochberg (BH) adjustment of the *p*-value. The statistical results showed no difference in the expression levels of the same genes between HbSS patients with and without retinopathy (Figures 4A–F; Supplementary Figure S4). In addition, low levels of expression for the *CCL2, CXCL10, GBP5* and *TGFB2* genes were observed in HbSC patients with PSCR, when compared with HbSC patients without PSCR. On the other hand, no difference was observed among HbSS patients with and without PSCR (Figures 5A–H; Supplementary Figure S4). The differential expressions of the up-regulated genes, *ROBO1* and *SLC38A5,* and the down-regulated genes, *CCL2*, *CXCL10*, *GBP5* and *TGFB2*, observed in qRT-PCR are consistent with the HbSC RNAseq data. However, unlike the RNAseq results, no significant difference was detected for the up-regulated genes, *PROX1*, *SEMA3B* (Figures 5G–J; Supplementary Figure S4), and the down-regulated gene, *OASL* (Figures 6I, J; Supplementary Figure S4), when comparing HbSC patients affected or unaffected by retinopathy by qRT-PCR. Moreover, these latter genes showed no difference in expression levels between groups III and IV.

## Discussion

PSCR is considered the most serious ocular complication of SCD, and can potentially result in visual loss due to vitreous hemorrhage or retinal detachment. PSCR is more frequently manifested in patients with HbSC than in other genotypes, and some degree of visual loss can occur in 5%–20% of affected eyes. The mechanisms involved in the higher incidence of ocular alterations in HbSC patients remain unclear. Considering that the neovascularization process involves the recruitment of endothelial progenitor cells, in the present study we compared, for the first time, the transcriptional profiles of ECFCs from patients with HbSC hemoglobinopathy and proliferative retinopathy versus patients without retinopathy. ECFCs, endothelial progenitor cells that circulate in peripheral blood, have a high proliferative capacity [32] and stable phenotype during *in vitro* culture and have been used as a tool for understanding the pathophysiology of vascular diseases and as a model for the study of endothelial function in sickle cell anemia [17, 33]. Emerging pre-clinical evidence underscores the therapeutic potential for ECFCs in ischemic retinopathies, such as diabetic retinopathy and retinopathy of prematurity, promoting vascular repair and revascularization [13–15].

RNA-seq was used to find DEGs, followed by functional enrichment analysis and PPI network, a common workflow in gene expression studies. The functional enrichment analysis for these DEGs demonstrated that they were significantly enriched in some GO terms that are associated with PSCR, including angiogenesis, cell migration, cell adhesion and innate immune response. Previous studies have demonstrated the important role of pathways associated with the immune system [34] and angiogenesis [35] in the pathogenesis of SCD. Interestingly, our results agree with these findings. Gene ontology analysis showed that the top significant biological terms derived from the list of DEGs included processes like vasodilation, type I interferon signaling, innate immunity and angiogenesis. Moreover, data derived from PPI network analysis reflected a similar pattern. Enrichment analysis of clusters showed that terms associated with type I interferon signaling, innate immune response, axon guidance and angiogenesis were also among the top ranked findings. Results were consistent across different analytical tools. The Cytohubba analysis of the main clusters (1–6) shows that the top six hub genes are *IFIT1*, *ITGB2*, *ROBO1*, *BDKRB2*, *CCL2* and *COL4A1*. The *IFTI1* and *ITGB2* genes play important roles in the immune response and *ROBO1*, *BDKRB2*, *CCL2* and *COL4A1* are associated with angiogenesis. Interestingly, interferon-associated molecular signals, including type I interferon signaling genes such as *IFIT1*, *OASL*, *OAS2*, *IFI6*, *XAF1*, *MX1* are all down-regulated in ECFC of HbSC patients presenting PSCR, when compared with HbSC patients unaffected by PSCR. Likewise, other genes associated with the immune system processes are also downregulated in the ECFC of these patients, such as *CEACAM1*, *CD300A*, *CTSS*, *ITGB2*, *SPI1*, *C1QA*, *LILRA5*, *LTB.*

Several studies, particularly in cancer, have demonstrated the anti-angiogenic activity of type I interferons. This activity has been attributed to various alternative mechanisms [36], including inhibition of basic fibroblast growth factor (*bFGF*) overproduction by tumor cells [37] or down-regulation of *IL-8* and *VEGF* gene expression [38, 39]. IFN-α seems to also have a direct effect on EC, including inhibition of EC proliferation and migration [40–42]. Additionally, IFNs induce the expression of many genes and it has been proposed that the antiangiogenic effects of type 1 interferons may be associated with the up-regulation of angiostatic chemokines, including *CXCL10*, and *CXCL11* [40]. In fact, there is abundant evidence to indicate that CXC chemokines are involved in inhibiting angiogenesis [43]. Guanylate-binding proteins (GBPs) are also a group of IFN-induced proteins that are essential for innate immune responses [44] and can mediate the inhibition of EC proliferation [42] and invasion [45]. The anti-proliferative activity of GBP-1 has been associated with decreased progression in some cancer types, such as breast and colorectal cancer [46]. High expression of *GBP1-5* has been correlated with favorable overall survival in skin cutaneous melanoma (SKCM) patients. In the present study, *CXCL10*, *GBP4* and *GBP5* are downregulated in the ECFC of HbSC patients with PSCR. Since our results revealed reduced expressions of all type I interferon signaling genes, we hypothesized that HbSC patients with PSCR may have an IFNα/β gene signature, which leads to failure in producing significant antiangiogenic effects, contributing to an efficient neovascularization process in these patients.

It is important to emphasize that, considering the notorious clinical heterogeneity of SCD, it is possible that the significantly enriched pathways and the associated genes identified in the analysis of SCR may also be involved in other complications present in the patients studied. In this regard, we highlight the avascular necrosis present in the HbSC patient group with retinopathy (3/6). Avascular necrosis (AVN), also known as osteonecrosis, is a common consequence of vaso-occlusive processes that affect the musculoskeletal system in patients with sickle cell haemoglobinopathy. There is established evidence that angiogenesis, the main pathway identified in this study, and bone formation work closely together in this complication of SCD [47]. The biology of osseointegration involves various processes including inflammation, vascularization, and bone formation [48]. Nevertheless, further studies are necessary to better understand the involvement of DEGs in the cascade of molecular processes that help manage the physiopathology of this complication.

Studies have increasingly indicated that the development of the nervous and vascular systems share similar guidance mechanisms [49, 50]. Among the guidance systems involved in axonal and vascular networks, there are the Slits and Roundabouts (ROBO), Netrins and UNC5 receptors, Semaphorins, and Eph receptors and ephrin ligands. Despite this great complexity of pathways and these already known guidance molecules signaling, interestingly, we identified remarkable overexpression of the *ROBO1* gene in the ECFCs of SC patients with PSCR (log2FoldChange = 4.3, FDR = 1.35E-11) indicating that most likely this receptor is crucial for angiogenesis in sickle cell retinopathy.

ROBO1, a member of the ROBO family, plays key roles in axon guidance receptor regulation and has been reported to mediate tumor angiogenesis [51]. Robo1 may play an important role in cell tube formation, cell attachment, proliferation and promote migration of endothelial cells [51, 52]. Previous studies using monkey choroidal retinal endothelial cells [52], HUVECs [51] and mouse retinal endothelial cells [53] have demonstrated that decreased Robo1 inhibited EC migration. Previous researchers have suggested that *Robo1* is involved in ocular neovascularization. Data show significantly elevated expression levels of Robo1 in the retinas of mice with oxygen-induced retinopathy of prematurity [52–54]. ROP is characterized by local ischemia and preretinal neovascularization. In a comprehensive study, Rama et al. provide clear genetic evidence for the crucial role of Slit2 signaling through Robo1 and Robo2 for promoting pathological and developmental ocular neovascularization [54]. In addition, it has been reported that sprouting angiogenesis, induced by VEGF-A, also requires the presence of Robo1. In the present study, the significant overexpression found in the ECFCs of HbSC patients with PSCR suggests that Robo1 may play a potential role in the pathophysiology of sickle cell retinopathy.

Among the differentially expressed genes identified, we also highlight the *SLC38A-5* gene (solute carrier family 38, member 5), which encodes an amino acid transporter expressed mainly in Muller cells, ganglion cells and endothelial cells [55]. We found that *SLC38A5* is significantly up-regulated (log2FoldChange = 3.4, FDR = 1,59E-07) in the ECFCs of SC patients with PSCR. *SLC38A5* is a neutral amino acid transporter responsible mostly for glutamine uptake in the retina.

The metabolism of ECs has been recognized as a regulator of angiogenesis and the concept that targeting EC metabolism may represent a novel anti-angiogenic strategy to treat vascular disease is emerging [56, 57]. A growing amount of evidence indicates that endothelial cell glycolysis, fatty acid oxidation and glutamine metabolism are essential in the angiogenic behavior of ECs during vessel formation [58, 59]. Glutamine is the most abundant nonessential amino acid in the human body and contributes to every biosynthetic pathway in proliferating cells [60]. Glutamine is a key carbon and nitrogen source for nucleotide and protein synthesis. Additionally, once transported into the cells, glutamine can be converted to glutamate followed by conversion to ornithine to generate polyamines and nitric oxide (NO), both pro-angiogenic factors [56].

The role of glutamine metabolism in cancer cells has been well characterized. The increased influx of glutamine in cancer is associated with the up-regulation of transporters to satisfy the increased demand for amino acids due to rapid cellular growth and proliferation [60, 61]. Computational analysis focusing on the gene expression data for glutamine and glutamate metabolism in different cancer types showed that the uptake of glutamine is increased by up-regulated *SLC38A5* in breast invasive carcinoma (BRCA), colon adenocarcinoma (COAD), and head and neck squamous cell carcinoma (HNSC) [62]. On the other hand, the role of glutamine metabolism in ECs during angiogenesis is not well known. Nonetheless, studies have demonstrated that depriving ECs of glutamine or inhibiting glutaminase 1 (GLS1) causes impaired EC proliferation, migration and vessel sprouting *in vitro* and *in vivo* [63, 64]. It was reported that endothelial loss of GLS1 diminishes the number of vascular branch points of the vascular plexus and reduces radial expansion of the plexus in the retina of EC-specific GLS1 knockout mice. In addition, pharmacological blockage of glutamine metabolism reportedly reduces pathological ocular angiogenesis in mouse pups with retinopathy (ROP model), which may imply a possible new antiangiogenic strategy [63, 64].

Additionally, in this study we also performed expression level analysis for the ten DEGs in the HbSS samples, with and without retinopathy (groups III and IV). Interestingly, the difference in expression was observed only for the *TGFB2* gene. Therefore, our results indicate that different genes may be involved in sickle cell retinopathy of HbSC and HbSS hemoglobinopathies. We previously employed the present approach to reveal gene expression differences between HbSS patients with and without PSCR [65]. In fact, different sets of genes have been identified coincident with a corresponding increase in endothelial cell expression associated with cell migration, cell adhesion and proliferation signaling. However, further studies with different approaches are necessary to confirm this hypothesis.

This study has some limitations, such as the small number of patients in each group and the lack of quantification of the protein levels of the DEGs. Small sample sizes are not rare in studies employing RNAseq in human samples [66, 67], and can influence the risk of false positives or false negatives results. Additionally, in the present study, five patients (one HbSC patient and four HbSS patients) were undergoing regular blood transfusion. Red blood cell transfusion remains an important therapeutic intervention for improving oxygen carrying capacity and reduce the complications of vaso-occlusion associated with sickle cell disease. Regarding the transfusion treatment and consequent effect on gene expression in circulating ECFCs, it is important to highlight that the peripheral blood samples for isolation of ECFCs were collected immediately before the transfusion of red blood cells. In addition, previous validation study of the present approach demonstrated that alterations in ECFCs gene expression profile as consequence of cell culture stimulation with inflammatory cytokines returned to baseline after one expansion [68]. Since cells from passages 3 to 5 were used for RNA sequencing in our study, we believe that the acquired endothelial phenotypes due transfusion treatment may have been washed out and that therefore, the gene expression of ECFCs reflects only genetic profile of the patients.

Moreover, it is important to emphasize that our approach was based on cultured ECFCs, which are not endothelial cells that reside naturally in the retina. Hence, they are free of tissue specification phenotypes and acquired influences. However, studies have shown that ECFCs demonstrate functional capacity *in vitro* and *in vivo* to both form vessels and integrate into pre-existing vasculature. In this way, evidence from pre-clinical investigations indicates that the administration of ECFCs may present therapeutic efficacy by promoting vascular repair in ischemic disorders, such as ischemic retinopathy [14] and ischemic brain [69]. Therefore, we believe that our strategy has generated data that may provide novel insights for understanding the pathophysiology underlying HbSC sickle cell retinopathy, which remains poorly comprehended.

In conclusion, the results shown here reveal candidate genes involved in HbSC proliferative sickle cell retinopathy, of which we highlight the *ROBO1* and *SLC38A5* genes, which have been previously associated with retinopathy in an animal model and deserve more attention as an alternative strategy to inhibit pathological angiogenesis.

## Data Availability

The datasets presented in this study can be found in online repositories. The names of the repository/repositories and accession number(s) can be found in the article/Supplementary Material.

## Footnotes

1
https://sankeymatic.com/
