# Supplementary material for: The molecular mechanism responsible for HbSC retinopathy may depend on the action of the angiogenesis-related genes ROBO1 and SLC38A5
Source: Exp Biol Med (Maywood). 2024 Jul 24;249:10070. doi: 10.3389/ebm.2024.10070 (PMC11303203; doi:10.3389/ebm.2024.10070)
Supplement: Supplementary file 1 [file DataSheet1.pdf]

Supplementary Table 1: Demographic data and hematological parameters of HbSC patients.

Patients	Sex	Age * (Years)	RBC (10^6 /µL)	Hb (g/dl)	WBC (x10^9/L)	Reticulocytes (AN)	Platelets (x10^9/L)	HbF (%)	HbS (%)	HbC (%)	Alpha thalassemia
**With PSCR**											
1	F	37	4.22	11.5	14.66	7.68	486	06	47.8	45.6	Normal
2	F	57	3.92	10.7	4.32	5.65	140	03	43	42.7	Normal
3	M	46	4.34	13.1	7.25	5.82	211	03	50.4	43	Normal
4	M	35	4.73	13.3	7.22	5.24	341	08	48	43	Heterozygous
5	F	68	3.78	11.1	7.02	5.27	195	2.4	45.1	45.1	Normal
6	F	64	3.75	11.3	5.77	7.96	388	0.5	49.8	43.1	Normal
**Without PSCR**											
7	F	52	4.16	11.4	10.87	5.56	508	2.0	47.0	44.4	Normal
8	F	35	3.81	11.6	11.78	9.41	242	2.2	47.5	43.8	Heterozygous
9	F	39	3.95	11.9	9.89	4.14	333	1.7	48.4	43.4	Normal
10	M	42	4.64	12.5	8.45	7.3	366	0.5	48.2	43.8	Heterozygous

*Age at the time of collection, RBC: Red blood cell count, WBC: White blood cell count, AN: Absolute numbers, Hb: Hemoglobin, HbF: Fetal hemoglobin, HbS: Hemoglobin S, HbC: Hemoglobin C.

Supplementary Table 2: Demographic data and hematological parameters of HbSS patients.

Patients	Sex	Age (Years)*	RBC (x10^6/µl)	Hb (g/dl)	WBC (x10^3/µl)	Reticulocytes (AN)	Platelets ($x10^9$/L)	HbF (%)	HbS (%)	Alpha thalassemia
**WITH PSCR**										
1	M	43	2.8	8.5	12.6	6.2	371	1.4	14.4	Normal
2	F	34	2.7	7.4	8.6	11.2	420	3.1	90.3	Heterozygous
3	F	29	3.7	9,2	6.1	16.9	383	3.3	88	Heterozygous
4	F	28	3.6	11.4	9.4	14.5	436	2.8	43.3	Normal
**WITHOUT PSCR**										
5	F	26	2.8	8.9	11.2	16.1	473	1.3	24.1	Normal
6	M	39	3.0	9.6	11.1	9.5	285	9.5	84.6	Normal
7	F	55	2.4	8,2	7.8	7.3	299	2.8	11.2	Normal
8	F	29	2.3	7.9	8.2	12.4	433	11.4	85	Normal

*Age at the time of collection, RBC: Red blood cell count, WBC: White blood cell count, AN: Absolute numbers, Hb: Hemoglobin, HbF: Fetal hemoglobin, HbS: Hemoglobin S.

Supplementary Table 3: Clinical data, treatment, and ophthalmic characteristics of HbSC patients.

Patients	Clinical data		Treatment
	**Ophthalmic signs**	**Other complications**	
1	Proliferative Retinopathy<br>Fibrous proliferation with traction in periphery of temporal retina of the right eye; photocoagulation scars in inferior temporal periphery of the right eye; neovessels in sea fan in temporal retina of the left eye; black sunburst in superior temporal quadrant of the left eye.	Avascular necrosis of the femoral head.	Enoxaparin sodium,<br><br>Folic acid, vitamin D, $CaCO_3$.
2	Proliferative Retinopathy<br>Neovessels in temporal retina in both eyes; area of retina traction with fibroglial tissue in the inferior temporal vascular arch of the left eye.	Absence of clinical complications.	Folic acid.
3	Proliferative Retinopathy<br>Regressed neovessels; photocoagulation scars in temporal retina in both eyes.	Absence of clinical complications.	Folic acid, vitamin D, $CaCO_3$.
4	Proliferative Retinopathy<br>Neovessels in sea fan in temporal retina of the left eye.	Cholecystectomy secondary to gallstone, renal Lithiasis.	Folic acid, vitamin D, $CaCO_3$.
5	Proliferative Retinopathy<br>Photocoagulation scars in mid-periphery; drusen and retinal pigmented epithelium (RPE) atrophy in macular region.	Ischemic stroke, nephropathy, avascular necrosis of the femoral head.	Regular transfusion, captopril and propranolol, Simvastatin, Folic acid, vitamin D, $CaCO_3$.
6	Proliferative Retinopathy<br>Fibrovascular proliferation in macular region in the right eye and in superior temporal arch in the left eye; epiretinal membrane and full thickness macular hole in the left eye.	Cholecystectomy, avascular necrosis of the femoral head, osteoporosis.	Folic acid, vitamin D, $CaCO_3$, Alendronate.
7	No pathological signs.	Absence of clinical complications.	Folic acid, vitamin D, $CaCO_3$,<br>Omeprazole,,glucosamine sulfate, chondroitin sulfate, Alendronate.
8	No pathological signs.	Pre-eclampsia, cholecystectomy,	Folic acid, vitamin D, $CaCO_3$.
9	No pathological signs.	HELLP Syndrome, Avascular necrosis of the femoral head.	Folic acid, vitamin D, $CaCO_3$.
10	No pathological signs.	Nonclassical mitral valve prolapse.	Enalapril, folic acid.

Supplementary Table 4: Clinical data, treatment and ophthalmic characteristics of HbSS patients.

Patients	Clinical Data		Treatment
	**Ophthalmic signs**	**Other complications**	
1	Proliferative retinopathy; Fibrovascular proliferation in mid-peripheral retina of the right eye.	Leg ulcer; osteoporosis; pulmonary hypertension; chronic renal failure; chronic venous insufficiency; ischemic stroke.	Acetylsalicylic acid; sodium alendronate; $CaCO_3$; vitamin D; deferasirox; enalapril maleate; folic acid; transfusion.
2	Proliferative retinopathy; Neovessels in *sea fan* with fibrotic areas in superior retina of the right eye, cataract.	Hypothyroidism; cholecystectomy; pulmonary hypertension; mild mitral insufficiency, acute chest syndrome.	Folic acid; enalapril maleate; sodium levothyroxine.
3	Proliferative retinopathy; Retinal detachment in the left eye, photocoagulation in the right eye.	Leg ulcer.	Folic acid.
4	Proliferative Retinopathy; Photocoagulation in the left eye.	Cholecystectomy; discreet hepatomegaly; leg ulcer; mild mitral insufficiency with non-classic mitral valve prolapse; ischemic Stroke.	Acetylsalicylic acid; folic acid; injectable contraceptive; transfusion.
5	No pathological signs.	Cholecystectomy; mild mitral and aortic insufficiency; ischemic stroke.	Folic acid; desferasirox; $CaCO_3$; omeprazole; transfusion.
6	No pathological signs.	Erectile dysfunction; perforated duodenal ulcer.	Acetylsalicylic acid; folic acid.
7	No pathological signs.	Pain crisis; femoral avascular osteonecrosis; chronic hepatopathy; osteopenia; splenic infarction; chronic renal disease; cholecystectomy.	Enalapril maleate; omeprazole; folic acid, caco3 +vitamin D; sodium bicarbonate; doxycycline, transfusion.
8	No pathological signs.	Absence of clinical complications.	Folic acid.

Supplementary Table 5: Primer sequences used for real-time quantitative PCR (qRT-PCR) analysis.

Gene	5'- 3' Sequence	Annealing temperature (ºC)	Fragment Size (bp)
ROBO1	F: CCAAGGTGTAACTGTATCCAAGAAT	66.5	121
	R: CCCAGACACCAAACCTTATACTCT	66.2	
SLC38A5	F: TGAAACACTTGGGCTACCTGG	66.9	135
	R: CACTCTCCATTGCTGTTTCATTG	66.8	
NNAT	F: CATCATCGGCTGGTACATCTT	62	130
	R: GTACCTGAACACCTCACTTCTC	62	
PROX1	F: GCTCTCCTTGTCGCTCATAAA	62.0	91
	R: TCCTTCCTGCATTGCACTT	62.0	
SEMA3B	F: TCCTACAAGTTGGAGCCAATTAC	62	103
	R: CCAACGTCTGTGCCAATGA	63	
CCL2	F: GCTCAGCCAGATGCAATCAAT	62.6	110
	R: CACTTGCTGCTGGTGATTCTTC	62.3	
CXCL10	F: CCACGTGTTGAGATCATTGCTAC	61.8	121
	R: GAGATCTTTTAGACCTTTCCTTGCT	59.8	
GBP5	F: GGATATGGTGTGTGCCTCAT	62.0	96
	R: TCTTGTTGTCAGCCTTCTCTAC	62.0	
TGFB2	F: CATCGACAGCAAAGTTGTGAAA	62.0	105
	R: CCCAGGTTCCTGTCTTTATGG	62.0	
OASL	F: TTCTGAGGCAGGAGCATTTC	62.0	78 pb
	R: AGCCCACCTTGACTACCT	62.0	

Supplementary Table 6: List of up-regulated genes identified when comparing SC patients with and without PSCR considering statistically significant FDR < 0.1.

Gene Name	Log2FoldChange	p-value	FDR
ROBO1	4.32524924	7.14E-16	1.35E-11
SLC38A5	3.36425349	1.69E-11	1.59E-07
MEG8	2.88826884	1.05E-10	5.37E-07
EPS8	1.91204649	1.14E-10	5.37E-07
DDR2	2.78386266	4.55E-07	0.0006986
PEG3	2.77158318	5.18E-07	0.0006986
CACNG8	1.94340347	7.29E-06	0.00510006
PLPP7	2.58241867	9.67E-06	0.00612908
TRBJ2-1	2.57528902	1.04E-05	0.00612908
PLPP4	2.17071609	1.61E-05	0.00870979
APCDD1L	2.40192799	1.92E-05	0.00929065
SEMA3B	2.04918836	2.14E-05	0.00984588
LOC100132077	2.16918088	2.29E-05	0.01028947
FRG1DP	2.32094269	2.83E-05	0.01189039
RIMS1	2.41002147	3.84E-05	0.01510777
CLMP	2.38543365	4.18E-05	0.01578749
PROX1	2.29249045	5.32E-05	0.01929934
LINC01291	2.33977052	6.51E-05	0.02317946
TRBC2	2.31764971	7.10E-05	0.02439142
COL8A2	1.58538383	7.11E-05	0.02439142
GFPT2	2.28440348	7.45E-05	0.02467624
ERC2	2.21061772	8.49E-05	0.02715476
INPP4B	1.72026521	0.0001073	0.03116652
DUSP4	1.72612389	0.00013481	0.03534889
SLC1A1	1.71029082	0.00014464	0.03740867
CCDC152	2.1601915	0.0001596	0.03979575
NNAT	2.01434507	0.00017696	0.04283267
IGF2BP1	2.15579992	0.00020844	0.04799232
TRBC2	2.11028058	0.00030685	0.06366281
TWIST2	2.09236618	0.00032548	0.06599271
AC078850.1	1.9968279	0.00053746	0.08627703
PRKG1	1.93927518	0.00055681	0.08627703
DSP	1.99607881	0.00064624	0.09683342
MALAT1	1.95983371	0.00065419	0.09725317
ACTN2	1.98100483	0.0006857	0.09862665
HIF1A-AS3	1.94906211	0.00066881	0.09862665

Supplementary Table 7: List of down-regulated genes identified when comparing SC patients with and without PSCR considering statistically significant FDR < 0.1.

Gene Name	Log2FoldChange	p-value	FDR
ZNF365	-3.1690819	6.43E-08	0.00020221
OAS2	-2.2534868	6.32E-08	0.00020221
CHRNA1	-2.4937386	2.02E-07	0.00047686
ADRB1	-2.3931469	1.83E-07	0.00047686
CMPK2	-2.713139	3.06E-07	0.00064209
TGFB2	-2.8376845	3.70E-07	0.00069772
MAF	-2.2824508	4.63E-07	0.0006986
BDKRB2	-2.0182353	5.04E-07	0.0006986
LGALS3BP	-2.2819744	1.44E-06	0.00180634
TSPAN2	-2.5301883	1.69E-06	0.00199874
OASL	-2.4472849	1.85E-06	0.00205033
FAXC	-2.6739932	1.98E-06	0.00207921
SUSD5	-2.363091	3.69E-06	0.0036662
CD300A	-2.6915666	4.37E-06	0.00395282
GBP4	-1.9038406	4.40E-06	0.00395282
CXCL10	-2.6270654	5.40E-06	0.00463322
CD101	-2.6554814	5.85E-06	0.00468423
EPSTI1	-1.850793	5.95E-06	0.00468423
GBP5	-2.5271787	7.28E-06	0.00510006
CCL2	-2.0615109	6.83E-06	0.00510006
CCDC178	-2.0437119	7.71E-06	0.00519842
MX1	-2.2128911	1.01E-05	0.00612908
MSX1	-1.8677715	1.00E-05	0.00612908
JAK3	-1.7561169	1.26E-05	0.0071874
BVES	-1.9445916	1.35E-05	0.00748557
CYTL1	-2.4995743	1.73E-05	0.00909414
CTSS	-1.8473701	1.79E-05	0.00913622
CPA3	-2.2429602	1.92E-05	0.00929065
MIR3142HG	-1.8804088	2.01E-05	0.00947905
SERPINA5	-2.2434085	2.37E-05	0.01039026
CCND2	-2.4420566	2.50E-05	0.01074555
SOD2	-1.0005573	3.11E-05	0.01275183
IFI6	-1.9436703	3.31E-05	0.01331526
KIAA1217	-1.9163272	4.13E-05	0.01578749
HHAT	-1.5068048	4.95E-05	0.01832387
GYPC	-1.5054628	7.39E-05	0.02467624
RGS9	-1.9009477	7.66E-05	0.02493033
IFIT1	-1.8413549	8.66E-05	0.02726494
SRPX2	-1.1817992	9.59E-05	0.02969109
UNC5A	-2.2086131	0.0001038	0.03062137
CREG2	-2.0884723	0.00010301	0.03062137
LARGE1	-1.4937984	0.00010252	0.03062137
AL121749.1	-2.2071057	0.00011198	0.03200435
HCP5	-2.1499244	0.00011357	0.03200435
MAMDC2	-1.9723632	0.00011561	0.03209977
ABCA4	-1.1514631	0.00012376	0.03386392
PCDH1	-1.4767424	0.00013094	0.03531758
RCSD1	-2.2195092	0.00013317	0.03534889

TPD52L1	-2.0162585	0.00014944	0.03812782
LILRA5	-2.1859132	0.0001623	0.03979575
TRPV2	-1.9272475	0.00016135	0.03979575
C1QA	-2.1392412	0.00019779	0.04673945
ITGB2	-2.062284	0.00019805	0.04673945
TNFRSF10C	-1.6111712	0.00020236	0.04716717
PTPRN2	-1.9159104	0.00021976	0.0499884
CDKN2A	-1.758302	0.0002397	0.05387607
VAT1L	-2.0929131	0.00024377	0.05414512
GRAP2	-2.1021553	0.0002687	0.05898935
PRR26	-2.0729899	0.00028293	0.06070239
NUAK2	-1.7281788	0.00028024	0.06070239
NEURL3	-2.1126228	0.00028749	0.06098745
RIPK3	-1.5116095	0.00029381	0.06163463
N4BP3	-1.5467122	0.00031262	0.06415608
LTB	-1.5687443	0.00033206	0.06599271
CXorf36	-1.1057838	0.0003286	0.06599271
ABCA1	-1.5552026	0.00034732	0.06819154
TMEM217	-1.5217457	0.00035035	0.06819154
LOC285629	-2.0782955	0.00037694	0.07261818
FHOD3	-2.0743963	0.00040398	0.07551676
TNFRSF4	-2.0614496	0.00040363	0.07551676
EPHB1	-1.9434226	0.00040324	0.07551676
PLEK	-1.9416013	0.00040849	0.07561043
POU2F2	-1.3870416	0.00041263	0.0756358
XAF1	-1.7750297	0.00042779	0.07766053
GBX2	-1.7203327	0.00043349	0.07794568
TGFBI	-1.9153664	0.00045475	0.08007221
GRIK4	-1.8628438	0.00045457	0.08007221
COL4A1	-1.6692257	0.00045804	0.08007221
GNA14	-1.3851032	0.00047	0.0814096
ZBED6CL	-2.040536	0.00049173	0.0829834
CEACAM1	-1.3965364	0.00048933	0.0829834
EFNB1	-1.1838697	0.00049227	0.0829834
HOXA11-AS	-1.7825897	0.00051034	0.08526675
ERBB4	-1.9562934	0.00051659	0.08555385
SPI1	-2.0151617	0.00055751	0.08627703
TMEM155	-1.9553735	0.00054117	0.08627703
TIMP3	-1.8539274	0.00054152	0.08627703
LOC105377068	-1.8305412	0.00055145	0.08627703
ICAM1	-1.7203982	0.00053232	0.08627703
TAPBP	-1.1356476	0.00055354	0.08627703
AMPD3	-1.3318465	0.00057034	0.08683912
NEDD9	-1.1424247	0.0005691	0.08683912
HTR1D	-1.3363488	0.0006232	0.09412764
LCP2	-1.9766884	0.00068154	0.09862665
ANGPTL4	-1.7331121	0.00067674	0.09862665
ADAM19	-1.3325821	0.00068955	0.09862665
F2RL3	-1.6286466	0.00070152	0.09884133
C15orf52	-1.0720025	0.00069977	0.09884133

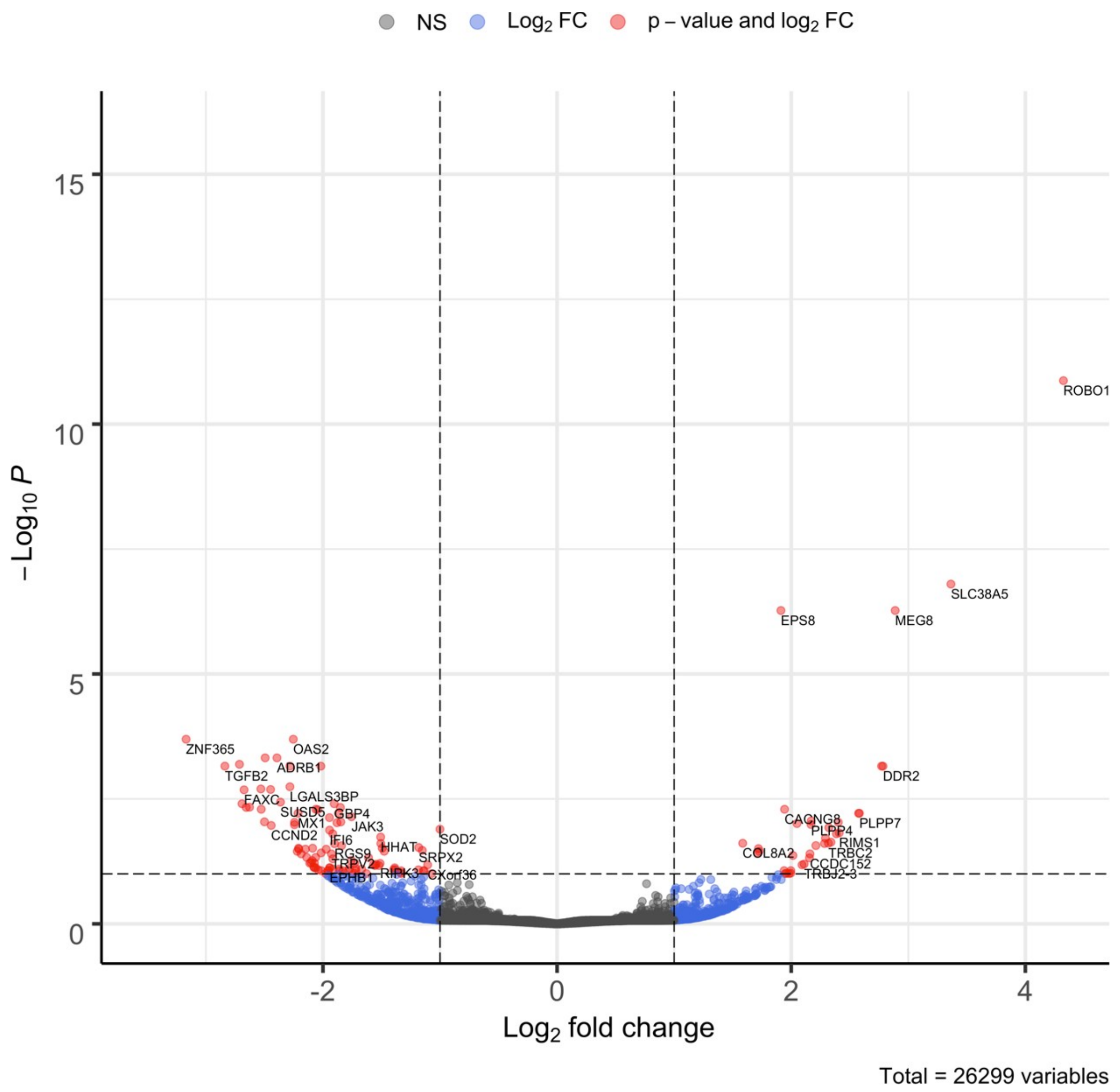

Supplementary Figure 1: Volcano plot of DEGs between HbSC patients with and without retinopathy. Each dot represents one gene with the y-axis showing $-\log_{10}$ p-value and the x-axis showing $\log_2$ (FC), respectively. Volcano plot shows downregulated (left) and upregulated (right) DEGs. The red dots represent significant DEGs with FDR <0.1 and log2FoldChange ≥| +1 ≤|-1|, in blue DEGs with $Log_2FC > 1$ and not significantly changed genes.

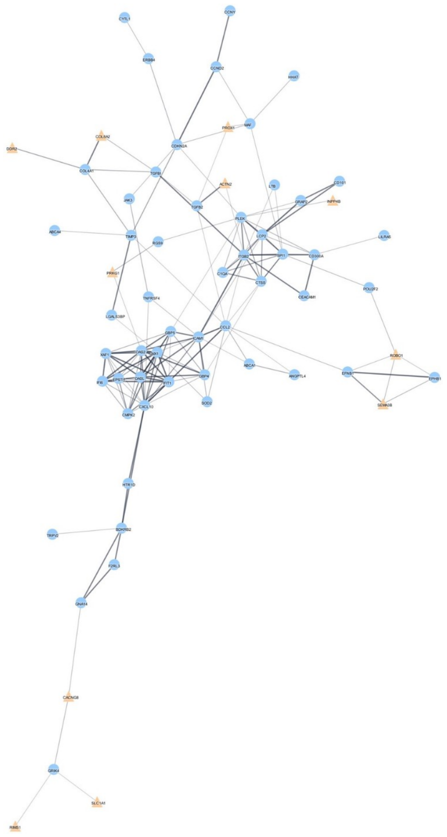

Supplementary Figure 2: Protein-protein interaction network constructed with the differentially expressed genes. Triangles represent up-regulated genes, and circles represent down-regulated genes.

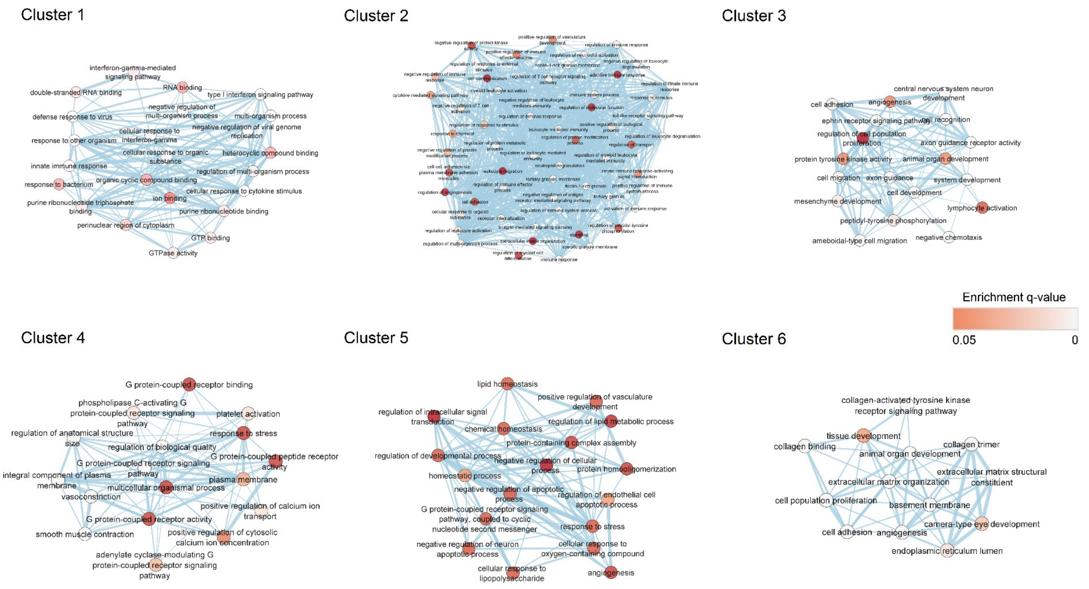

Supplementary Figure 3: GO enrichment analysis of each cluster, where node represents the functional group and edges represent term-term interaction. The significance of enrichment (q-value-FDR) is indicated by the color gradient.

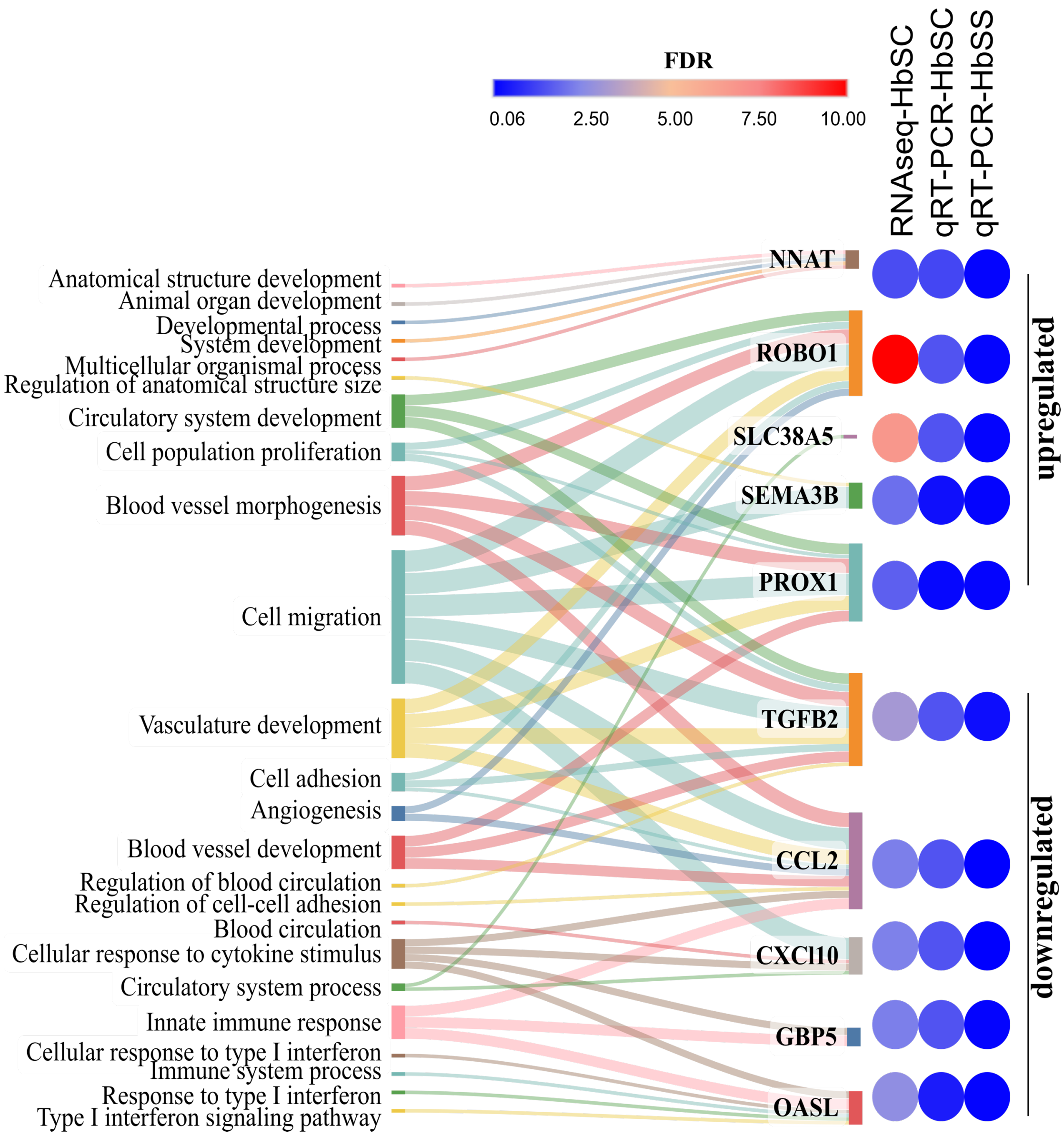

Supplementary Figure 4: Statistical analysis of RNAseq and qRT-PCR results of 10 DEGs selected for validation. Alluvial diagram showing the relationship between the main molecular pathways and enriched ontological terms for selected genes for validation, these analyzes were performed on Gene Ontology, and The Mann-Whitney test was used to calculate the p-values and the Benjamini Hochberg was used for the adjusted p-value (FDR).
